# Domain‐Specific and Domain‐General Functional Brain Alterations in Type 2 Diabetes Mellitus: A Task‐Based and Functional Connectivity Meta‐Analysis

**DOI:** 10.1002/brb3.70438

**Published:** 2025-03-31

**Authors:** Valentino Marcel Tahamata, Yang‐Teng Fan, Li Wei, Yen‐Nung Lin, Roger Marcelo Martinez, Kah Kheng Goh, Yu‐Chun Chen, Chenyi Chen

**Affiliations:** ^1^ Graduate Institute of Mind, Brain and Consciousness College of Humanities and Social Sciences Taipei Taiwan; ^2^ Graduate Institute of Injury Prevention and Control, College of Public Health Taipei Medical University Taipei Taiwan; ^3^ Graduate Institute of Medicine Yuan Ze University Taoyuan Taiwan; ^4^ Taipei Neuroscience Institute Taipei Medical University Taipei Taiwan; ^5^ Division of Neurosurgery, Department of Surgery Taipei Municipal Wan‐Fang Hospital Taipei Taiwan; ^6^ Department of Physical Medicine and Rehabilitation Taipei Municipal Wan‐Fang Hospital Taipei Taiwan; ^7^ School of Psychological Sciences National Autonomous University of Honduras Tegucigalpa Honduras; ^8^ Department of Psychiatry Taipei Municipal Wan‐Fang Hospital Taipei Taiwan; ^9^ Psychiatric Research Center Taipei Municipal Wan‐Fang Hospital Taipei Taiwan; ^10^ The Innovative and Translational Research Center for Brain Consciousness Taipei Medical University Taipei Taiwan; ^11^ Department of Physical Education National Taiwan University of Sport Taichung Taiwan

**Keywords:** functional decoding, neuroimaging meta‐analysis, resting‐state functional connectivity, Type 2 diabetes mellitus

## Abstract

**Introduction:**

Patients with Type 2 diabetes experience cognitive and affective deficits linked to widespread functional brain alterations. However, previous meta‐analyses of functional neuroimaging have primarily focused on the resting‐state studies. While valuable, this approach may have overlooked the key neuronal mechanisms underlying these deficits.

**Methods:**

To address this, we conducted a coordinate‐level task‐based meta‐analysis of functional neuroimaging via a systematic search of Embase, PubMed, Medline, and Web of Science (December 2023), alongside task‐based and task‐free connectivity analyses using the BrainMap database. Activation likelihood estimations were applied to two task categories: that are, cognitive and affective. To control for biased results, we also conducted jackknife sensitivity analysis and within‐paper combined experiments analysis as validation analyses.

**Results:**

We identified a cluster of activation in cognitive paradigm (18 contrasts, 153 foci, 767 subjects), with the middle frontal gyrus, part of the medial frontal cortex, as the peak region. For the affective paradigm (18 contrasts, 181 foci, 951 subjects), we observed both increased and decreased activities. Two increased clusters peak in the amygdala and middle temporal gyrus, while the three decreased clusters were inferior frontal gyrus, insula, and putamen. Follow‐up connectivity analyses showed that these brain alterations were both task‐specific and task‐generic.

**Conclusion:**

Despite the lack of uniformity across task, such domain‐specific and domain‐general patterns of alterations provide a more nuanced understanding of the cognitive and affective deficits in Type 2 diabetes patients. This highlights the varied neuronal mechanisms underlying these deficits.

## Graphical abstract

We conducted a task‐based functional neuroimaging meta‐analysis, identifying increased activity for cognitive tasks and both increased and decreased activity for affective tasks among Type 2 diabetes patients. Task‐based and task‐free connectivity analyses further revealed domain‐specific and domain‐general alterations, providing insights into the neural mechanisms underlying patients' cognitive and affective impairments.

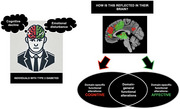

## 1 Introduction

Type 2 diabetes mellitus is a chronic metabolic disease characterized by elevated blood sugar levels that pose a global threat (Zheng et al. 2018). Type 2 diabetes patients exhibit insulin resistance or insufficient insulin production, leading to long‐term complications in various organs, including the brain (Manschot et al. 2007). Extensive literature links Type 2 diabetes to an increased risk of dementia and impaired cognitive functions (Strachan et al. 2011), with these cognitive deficits being associated with functional brain alterations (Zhou et al. 2014). In addition, a significant body of studies has reported an increased likelihood of emotional problems, such as distress and depression, among Type 2 diabetes patients (Snoek et al. 2015). Consequently, these multifaceted psychological deficits may lead to the overall reduction in the quality of life among Type 2 diabetes patients (C. Y. Huang, Lai et al. 2016; R. R. Huang, Jia et al. 2016).

One way to investigate the link between functional brain alterations and adverse psychological effects of Type 2 diabetes is by converging brain activities across functional neuroimaging studies involving Type 2 diabetes patients. A functional magnetic resonance imaging (fMRI) meta‐analysis revealed the convergence of altered brain regions, including the lingual gyrus, postcentral gyrus, inferior temporal gyrus, cerebellar culmen, insula, and posterior cingulate cortex (PCC; Xia et al. 2017). By applying a more‐stringent threshold, a recent meta‐analysis found consistent discovery of decreased activity in the right Rolandic operculum, right supramarginal gyrus, and right superior temporal gyrus (STG) across selected studies (Li et al. 2022). Some of these altered regions are key components of the default mode network (DMN), which is significantly associated with high‐level cognitive functions, including learning and memory.

Interestingly, a multimodal neuroimaging meta‐analysis (Yao et al. 2021) found conjoint (i.e., both structural and functional) convergence of brain alterations in DMN regions. However, that analysis also revealed decreased global (i.e., whole brain) gray matter volume (GMV) and a local GMV reduction in the limbic system without significant functional alterations. These regions are associated with cognitive functions and also emotion and motivation. The inconsistent convergence of activation patterns may be driven by the imaging techniques used in selected studies. It is important to note that the aforementioned meta‐analyses only included resting‐state neuroimaging studies, which might explain why the convergence of functional alterations was localized in the DMN or other resting‐state network areas, whereas structural alterations occurred widely across the brain, including subcortical areas (Antal et al. 2022). Furthermore, other functional neuroimaging studies investigated potential deficits related to emotional and motivational domains among Type 2 diabetes patients, such as emotion inhibition (Y. H. Chen et al. 2023) and the reward system (ten Kulve et al. 2015). Those studies were task‐based fMRI, where brain activations related to the functions they investigated would unlikely be captured via resting‐state imaging. It is worth noting that resting‐state and task‐based functional imaging modalities serve different purposes, with resting‐state brain activation traditionally associated with goal‐directed cognition (Spreng 2012). Therefore, including resting‐state studies in a meta‐analysis of Type 2 diabetes‐caused brain functional alterations might either confine the focus of alterations solely to “default” active areas during rest or, even worse, lead to potential fallacies when inferring associations between resting‐state activation and mental processes (Zhuo et al. 2021).

Hence, to obtain a more‐nuanced understanding of functional alterations in Type 2 diabetes and their implications in various deficits across psychological domains, we converged brain activation across task‐based functional neuroimaging studies, categorizing the tasks as either cognitive or affective. We then explored how these regions are functionally connected based on a large‐scale database. Finally, we examined whether these connections also appear when the brain is at rest. Together, these steps helped us uncover both specific and shared brain network changes linked to Type 2 diabetes.

## 2 Methods

As previously introduced, we performed a comprehensive approach to understand task‐based functional alterations in Type 2 diabetes. First, we conducted a meta‐analysis of task‐based functional neuroimaging studies to identify consistent activation patterns across cognitive and affective tasks using activation likelihood estimations (ALEs; Eickhoff et al. 2009). Second, we applied a large‐scale database meta‐analytic connectivity modeling (MACM) to examine task‐based functional connectivity, using the regions identified in the meta‐analysis to decode their coactivation patterns and associated psychological functions (Laird et al. 2013; Huang, Jia et al. 2016; Eickhoff et al. 2016). Third, we conducted a task‐free‐based analysis or the resting‐state functional connectivity (RSFC) of these regions in healthy individuals, providing insight into their intrinsic functional organization and potential relevance beyond task‐related activation. We describe these analyses in detail below. Finally, we performed this task‐based meta‐analysis on functional brain alteration in Type 2 diabetes in compliance with the Preferred Reporting Items for Systematic Reviews and Meta‐Analyses (PRISMA) statement, updated in 2020 (Page et al. 2021).

1 **Summary**

- Type 2 diabetes leads to widespread changes in brain activation patterns, and relying solely on resting‐state studies may limit the understanding of these changes.
- A task‐based coordinate‐level meta‐analysis on Type 2 diabetes revealed more extensive brain alterations, encompassing both cognitive and affective aspects, compared to resting‐state analyses.
- These findings are further supported by both task‐based and resting‐state connectivity analyses.

### 2.1 Literature Search and Selection

**FIGURE 1:**
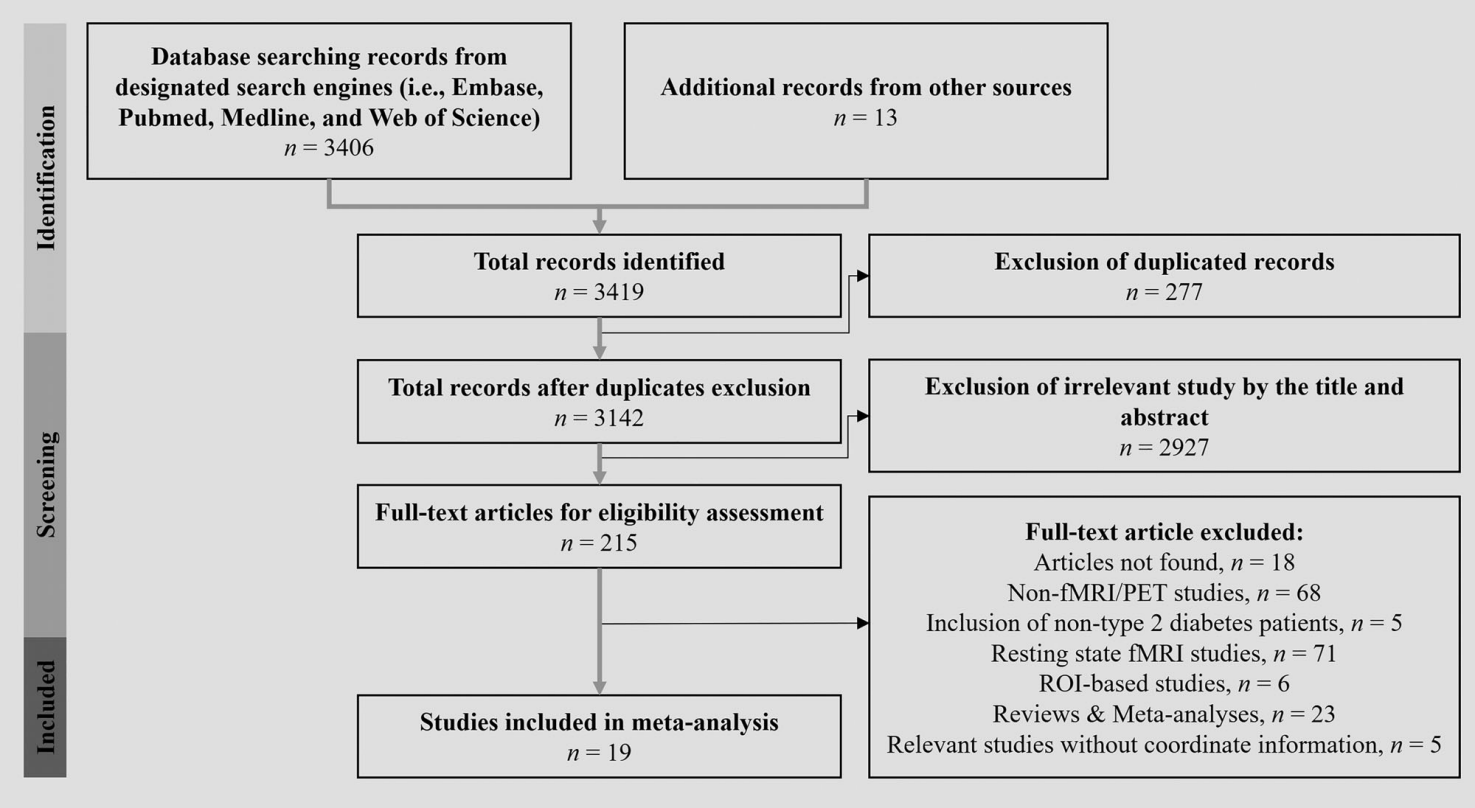
Flow chart of the study selection process.

To systematically search for relevant studies on this topic in December 2023, several online literature databases were utilized, including Embase, PubMed, Medline, and Web of Science. The search strategy involved two categories of keywords to ensure a comprehensive search: (“type 2 diabetes” or “diabetes mellitus, type 2” or “diabetes mellitus, type II”) and (“brain imaging” or “functional brain imaging” or “functional neuroimaging” or “fMRI” or “functional magnetic resonance imaging” or “positron emission tomography”). In addition, direct searches were conducted based on the keywords mentioned above. Identified records were then assessed based on the following criteria: (i) a paper reporting the use of an in‐scanner behavioral task, (ii) the functional imaging modality or technique capable of reporting clear source‐localized brain activation (e.g., fMRI, positron emission tomography (PET), or electroencephalography (EEG) with a low‐resolution brain electromagnetic tomography (LORETA) analysis), (iii) brain activations presented in standardized stereotactic space (i.e., Montreal Neurological Institute (MNI) or Talairach), (iv) the study including general linear model‐based whole brain coverage, and (v) contrasts derived from a general linear model based on either a binary contrast (e.g., Type 2 diabetes > healthy controls or target > control conditions) or a continuous parametric analysis. It is important to note that in this meta‐analysis, Talairach coordinates were converted into the MNI space using the icbm2tal algorithm (Lancaster et al. 2007). For the selection process, two reviewers (V.M.T. and C.C.) independently assessed each record and report for inclusion according to predetermined criteria. Initially, titles and abstracts were screened separately by both reviewers, followed by a full‐text review for studies that appeared eligible. Any discrepancies between the reviewers were addressed through discussion or, if necessary, by involving a third reviewer (Y.T.F.). No automation tools were employed during the screening or selection process. The final selection included 19 articles, with 18 total contrasts in cognitive and affective paradigms respectively (Figure 1, Table 1).

**TABLE 1:** Descriptive summary of included studies.

Study	Task category	Group, *N* participant, mean ± SD of age	Duration of T2DM (mean ± SD)	Imaging method	Tesla
Allen et al. (2016) (1)	Affective	**T2DM (15)** = 16.08 ± 1.53 years **Obese normal (21)** = 14.89 ± 1.98 years **Normal weight (21)** = 16.0 ± 1.91 years	35.8 ± 30.7 months	fMRI	3T
Backeström et al. (2021) (2)	Cognitive	**T2DM (36)** = 66.0 ± 2.20 years **Control (34)** = 65.8 ± 0.82 years	7.09 ± 4.17 years	fMRI	3T
Chechlacz et al. (2009) (3)	Affective	**T2DM (11)** = 55.36 ± 14.94 years **Control (12)** = 46.58 ± 15.58 years	6.3 ± 4.3 years	fMRI	1.5T
Duarte et al. (2015) (4)	Cognitive	**T2DM (51)** = 59.73 ± 7.90 years **Control (29)** = 56.67 ± 6.43 years	12.37 ± 8.76 years	fMRI	3T
Farr and Mantzoros (2018) (5)	Affective	**Obese T2DM (6)** = 49.40 ± 6.32 years **Non‐obese T2DM (5)** = 46.50 ± 4.09 years	No information	fMRI	3T
Frank et al. (2016) (6)	Affective	**T2DM with RYGB (12)** = 50.0 ± 2.67 years **T2DM without RYGB (12)** = 50.7 ± 3.29 years	**T2DM with RYGB** = 11.17 ± 1.91 years **T2DM without RYGB** = 6.46 ± 1.09 years	fMRI	3T
Huang, Jia et al. (2016) (7)	Cognitive	**T2DM (18)** = 43.33 ± 6.41 years **Control (18)** = 43.17 ± 6.48 years	No information	fMRI	1.5T
Marder et al. (2014) (8)	Cognitive	**T2DM (22)** = 56.0 ± 5.5 years **Control (29)** = 52.7 ± 5.5 years	9.0 ± 6.3 years	fMRI	3T
McDermott et al. (2019) (9)	Affective	**ILI (125)** = 58.39 ± 6.9 years **DSE (107)** = 57.80 ± 6.2 years	**ILI** = 58.39 ± 6.9 years; **DSE** = 6.87 ± 7.44 years	fMRI	3T
Sun et al. (2017) (10)	Cognitive	**T2DM (18)** = 42.89 ± 7.01 years **Control (18)** = 40.33 ± 4.14 years	Newly diagnosed	fMRI	1.5T
ten Kulve et al. (2016a) (11)	Affective	**T2DM (20)** = 59.3 ± 4.1 years	No information	fMRI	3T
ten Kulve et al. (2016b) (12)	Affective	**T2DM (20)** = 56.3 ± 1.4 years **Control (20)** = 59.5 ± 0.9 years	7.8 ± 1.1 years	fMRI	3T
van Bloemendaal et al. (2014) (13)	Affective	**T2DM (16)** = 61.4 ± 1.5 years **Normal obese (16)** = 58.0 ± 2.1 years **Normal lean (16)** = 57.8 ± 1.9 years	7.0 years	fMRI	3T
van Bloemendaal et al. (2015) (14)	Affective	**T2DM (16)** = 61.4 ± 1.5 years **Normal obese (15)** = 57.6 ± 2.2 years **Normal lean (16)** = 57.8 ± 1.9 years	7.0 years	fMRI	3T
Wood et al. (2016) (15)	Cognitive	**T2DM (22)** = 59.5 ± 5.3 years **Control (22)** = 59.5 ± 5.3 years **T2DM: monozygotic (8)** = 61.5 ± 5.6 **Dizygotic (14)** = mean 61.5 ± 5.6 years]	10.1 ± 9.7 years	fMRI	3T
Zhang et al. (2016) (16)	Cognitive	**T2DM (20)** = 54.15 ± 8.78 years **Control (19)** = 51.58 ± 6.19 years	No information	fMRI	3T
Salem et al. (2021) (17)	Affective	**T2DM RGYB (16)** = 48.6 ± 14.4 years **T2DM VLCD (19)** = 46.2 ± 10.8 years	No information	fMRI	3T
Y. H. Chen et al. (2023) (18)	Cognitive	**T2DM (48)** = 51.4 ± 6.5 years **Control (30)** = 48.7 ± 8.8 years	7.8 ± 3.8 years	fMRI	3T
Baker et al. (2011) (19)	Cognitive	**T2DM (12) + prediabetic (11)** = 74.3 ± 6.3 years **Control (6)** = 74.4 ± 7.1 years	No information	PET	n.a.

Abbreviations: DSE = diabetes support education; EXE = exenatide; HC = healthy control; ILI = intensive life intervention; RGYB = Roux‐en‐Y gastric bypass; T2DM = Type 2 diabetes mellitus; VLCD = very‐low‐calorie diet.

### 2.2 Experimental Grouping

As previously mentioned, the included studies in the current meta‐analysis encompassed two main task paradigm categories, more‐classic cognitive tasks (e.g., *N*‐back and Stroop task) and affective‐related tasks (e.g., food‐related tasks). In terms of functional alterations, we categorized activations into two groups: increased and decreased, which could result from group‐based contrasts (i.e., Type 2 diabetes vs. a control group) or task‐based contrasts (e.g., higher vs. lower memory loads or target interventions vs. control interventions/placebo) (Tables 2 and S1).

**TABLE 2:** Grouping paradigm, task, and contrasts included in current meta‐analysis.

Study	Task	Contrasts	Group	*N* participant (*N* Foci)
***Cognitive increased***				
Backeström et al. (2021)	*N*‐back	Higher > lower cognitive load	T2DM, obese, and HC	70 (3)
Duarte et al. (2015)	Speed discrimination task	T2DM > HC	T2DM and HC	81 (4)
Huang, Jia et al. (2016)	*N*‐back	Higher > lower cognitive load	T2DM only	18 (10)
Marder et al. (2014)	Encoding	Encoding > control blocks	T2DM only	22 (8)
Marder et al. (2014)	Recognition	Recognition > control blocks	T2DM only	22 (9)
Sun et al. (2017)	Iowa gambling task	Decision making > control condition	T2DM only	18 (12)
Wood et al. (2016)	Incidental encoding task	Encoding > control condition	T2DM and HC	44 (18)
Y. H. Chen et al. (2023)	Emotional Stroop task	Incongruent > congruent condition	T2DM only	48 (14)
Y. H. Chen et al. (2023)	Emotional Stroop task	Incongruent > congruent condition	T2DM and HC	78 (2)
Baker et al. (2011)	Memory encoding	Encoding < control condition	T2DM and prediabetic	23 (7)
***Cognitive decreased***				
Huang, Jia et al. (2016)	*N*‐back	T2DM < HC	T2DM and HC	36 (31)
Marder et al. (2014)	Encoding	Encoding < control blocks	T2DM only	22 (5)
Marder et al. (2014)	Recognition	Recognition < control blocks	T2DM only	22 (4)
Marder et al. (2014)	Encoding	T2DM < HC, encoding	T2DM and HC	51 (2)
Marder et al. (2014)	Recognition	T2DM < HC, recognition	T2DM and HC	51 (2)
Wood et al. (2016)	Incidental encoding task	Encoding < control condition	T2DM and HC	44 (12)
Zhang et al. (2016)	*N*‐back	T2DM < HC	T2DM and HC	39 (4)
Chen et al. (2023)	Stroop task	Incongruent < congruent condition	T2DM and HC	78 (6)
***Affective increased***				
Allen et al. (2016)	Food‐related exposure	Food > non‐food	T2DM only	15 (36)
Allen et al. (2016)	Food‐related exposure	Food > non‐food	T2DM, obese, and HC	57 (30)
Chechlacz et al. (2009)	Food‐related exposure	Food > non‐food	T2DM and HC	23 (18)
Farr and Mantzoros (2018)	Food‐related exposure	Higher > lower desirable food	T2DM obese, and non‐obese	11 (2)
Frank et al. (2016)	Food‐related exposure	Nonsurgical > RGYB	T2DM only	24 (4)
McDermott et al. (2019)	Food‐related exposure	DSE > ILI	T2DM only	232 (3)
ten Kulve et al. (2016a)	Food‐related exposure	Food > non‐food	T2DM only	20 (6)
van Bloemendaal et al. (2014)	Food‐related exposure	High calorie > low calorie food	T2DM only	16 (10)
van Bloemendaal et al. (2014)	Food‐related exposure	Food > non‐food	T2DM and HC	32 (1)
van Bloemendaal et al. (2015)	Food‐related exposure	Chocolate taste > tasteless	T2DM only	16 (5)
Salem et al. (2021)	Food‐related exposure	Food > non‐food	T2DM only	35 (19)
***Affective decreased***				
Frank et al. (2016)	Food‐related exposure	Nonsurgical < RGYB	T2DM only	24 (11)
McDermott et al. (2019)	Food‐related exposure	DSE < ILI	T2DM only	232 (3)
ten Kulve et al. (2016b)	Food‐related exposure	T2DM < HC	T2DM and HC	40 (3)
van Bloemendaal et al. (2014)	Food‐related exposure	T2DM < obese HC	T2DM and obese HC	32 (2)
van Bloemendaal et al. (2015)	Food‐related exposure	Placebo < EXE	T2DM only	16 (12)

Abbreviations: DSE = diabetes support education; EXE = exenatide; HC = healthy control; ILI = intensive life intervention; RGYB = Roux‐en‐Y gastric bypass; T2DM = Type 2 diabetes mellitus.

### 2.3 Activation Likelihood Estimation Analysis

The ALE method was employed for all meta‐analyses using GingerALE v.3.0.2 (Eickhoff et al. 2009) accessed through BrainMap (https://www.brainmap.org/ale/). This method estimates the likelihood of consistent brain activation across all included studies. The ALE method relies on a list of activated foci reported in a standardized stereotactic space (MNI or Talairach) and the number of subjects included in each experimental condition. This approach helps control spatial uncertainty arising from variations in subjects and templates used in neuroimaging data (Eickhoff et al. 2009). It is worth noting that the ALE method treats reported foci not as single or independent points, but rather as spatial probability distributions centered at the respective coordinates. In the current meta‐analysis, the significance of ALE scores was determined using the 10,000 permutation test and the significance of cluster‐level inferences was corrected with a cluster‐level family‐wise error (cFWE) threshold of *p* < 0.05.

### 2.4 Task‐Based Connectivity Analysis: Meta‐Analytic Connectivity Modeling Analysis and Functional Decoding

We used MACM to investigate common patterns of activation across all clusters yielded from the ALE method. The MACM analysis helps identify functional connectivity of brain regions obtained from the ALE analysis, shedding light on how these regions interact with other brain areas across a wide range of neuroimaging studies (Bedini et al. 2023). MACM integrates data from thousands of neuroimaging studies available in the BrainMap database (http://www.brainmap.org/) (Laird et al. 2013). We first collected all studies from the BrainMap database using Sleuth (https://www.brainmap.org/sleuth/) that shared at least one focus of activation within a cluster we initially obtained from ALEs. We then extracted the coordinates and information about the experiments and subjects from those studies. Finally, to derive co‐activation patterns of those studies, all coordinates were synthesized using ALEs with a thresholding method similar to that explained above in the ALE section.

We then conducted functional decoding using data obtained from the BrainMap database. BrainMap provides information about behavioral domains involved in each experiment within each study. Subsequently, we quantified and characterized all behavioral domains, presenting a functional profile of each peak brain region using both forward and reverse inference approaches. Forward inference [*p* (Activation | Domain)] denotes the probability of brain region activity being associated with any behavioral domain or psychological process, whereas reverse inference [*p* (Domain | Activation)] denotes the probability of the presence of a behavioral domain when activation of a brain region is known (T. Chen et al. 2018).

### 2.5 Task‐Free Connectivity Analysis: Resting‐State Functional Connectivity Analysis of each Identified Region and a Hierarchical Clustering Analysis

We performed a task‐free connectivity analysis to further elaborate the connectivity of each region identified in our main ALE analysis. We included resting‐state fMRI data of 278 healthy participants from the Nathan Kline Institute‐Rockland Sample (NKI‐RS: http://fcon_1000.projects.nitrc.org/indi/enhanced/; Fukushima et al. 2018; Gu et al. 2019). In the current study, functional connectivity was based on the average blood oxygen level‐dependent (BOLD) signal of each region within each cluster identified from the main ALE analysis as seed regions. Voxel‐wise correlation coefficients were then transformed into Fisher's *z*‐scores and tested for consistency across subjects. The preprocessing procedure is available in the Supporting Information. We then applied a Ward hierarchical clustering analysis to characterize the RSFC pattern of each region in each cluster (Gu et al. 2019).

### 2.6 Validation Analyses

To validate our main ALE findings, we implemented two validation methods. First, a jackknife sensitivity analysis, also known as leave‐one‐out sensitivity analysis, was conducted (Yao et al. 2021). Here, for each fold, we performed ALE in each experimental paradigm (i.e., increased and decreased cognitive and affective conditions) while excluding one experiment at a time. Second, within‐paper combined experiments were used (Gu et al. 2019). In this method, we performed an ALE analysis on each experimental paradigm by combining all experiments within a paper. This method helps avoid experiment selection bias in a paper and ensures that the ALE outcome does not solely come from one study with multiple experiments.

## 3 Results

### 3.1 Convergence of Activations Across Included Studies: Main ALE Results

**FIGURE 2:**
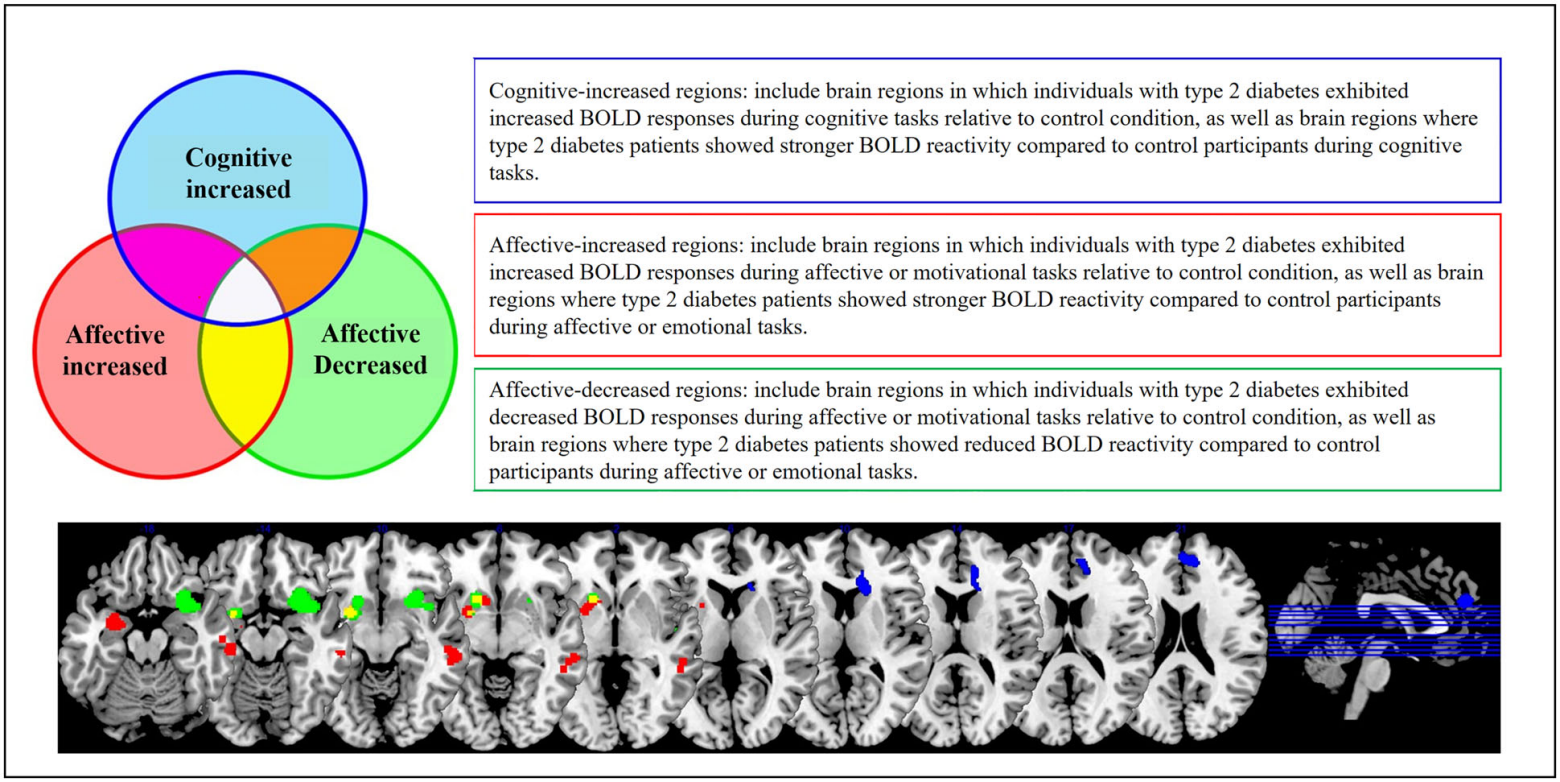
Convergence of brain activation across included studies. Blue‐colored areas depict the converged activations in cognitive‐increased paradigm, while areas with convergence of increased and decreased activation in affective tasks are colored in red and green, respectively.

We found significant convergence of brain activation in cognitive increased (10 contrasts, 424 subjects, 87 foci), affective increased (11 contrasts, 481 subjects, 134 foci), and affective decreased (five contrasts, 344 subjects, 31 foci) conditions, whereas no convergence of activation was found in the cognitive decreased (eight contrasts, 343 subjects, 66 foci) condition. For cognitive increased, 40% of contrasts contributed to a single cluster of activation in this condition, which peaked at the left medial frontal gyrus (MFG). For affective‐related paradigms, 30.36% of contrasts contributed to affective increased Cluster 1 (peak activation at the left amygdala), while 18.18% of contrasts contributed to affective increased Cluster 2 (peak activation at the right middle temporal gyrus [MTG]), and 60%, 40%, and 20% of contrasts respectively contributed to affective decreased Clusters 1 (peak activation at the right inferior frontal gyrus [IFG]), 2 (peak activation at the left insula), and 3 (peak activation at the left putamen). Please see Table 2 for detailed information on the number of participants and foci for each included study. In addition, we also performed a conjunction analysis between affective increased and affective decreased activations, which yielded two clusters of activation that respectively peaked at the left putamen and left caudate head (Figure 2, Table 3).

**TABLE 3:** Overall activation likelihood estimation (ALE) results on cognitive‐based and affective‐based task‐induced activation.

Cluster	Laterality	Brain regions	BA	MNI			Peak *z* Score	Cluster size (mm^3^)
				*x*	*y*	*z*		
**Cognitive increased activity**								
1	L	Medial frontal gyrus	9	0	50	26	4.01192	2360
	R	Medial frontal gyrus	9	10	46	20	3.56733	
	R	Caudate body	/	16	26	10	3.49446	
	R	ACC	32	14	38	14	3.09434	
**Cognitive decreased activity**								
None								
**Affective increased activity**								
1	L	Amygdala	/	−28	−2	−20	4.45813	3456
	L	Putamen	/	−24	6	−8	3.83122	
	L	Caudate head	/	−10	14	−6	3.17432	
	L	Putamen	/	−18	16	−4	3.0046	
	L	Putamen	/	−20	12	0	2.99874	
	L	Putamen	/	−24	12	4	2.71239	
2	R	Middle temporal gyrus	21	66	−24	−20	4.31091	2512
	R	Middle temporal gyrus	21	60	−32	−6	3.37025	
	R	Superior temporal gyrus	22	54	−24	−10	3.23712	
	R	Middle temporal gyrus	21	56	−34	−8	3.22776	
	R	Middle temporal gyrus	/	54	−34	−2	3.13475	
	R	Middle temporal gyrus	22	52	−40	−4	3.10826	
**Affective decreased activity**								
1	R	Inferior frontal gyrus	47	30	16	−16	5.2429	3648
	R	Claustrum	/	38	10	−14	3.9601	
2	L	Insula	13	−40	−8	16	3.4636	2040
	L	Insula	13	−44	−12	8	3.0942	
	L	Insula	13	−44	−18	8	3.0738	
3	L	Putamen	/	−18	14	−8	3.2117	1968
	L	Caudate head	/	−18	18	−4	3.0948	
	L	Putamen	/	−24	5	−11	3.0948	
	L	Putamen	/	−20	5	−11	3.0479	
**Affective increased ∧ affective decreased**								
1	L	Putamen	/	−24	5	−11	3.0948	496
2	L	Caudate head	/	−18	18	−4	3.0948	376

### 3.2 MACM and Functional Decoding

**FIGURE 3:**
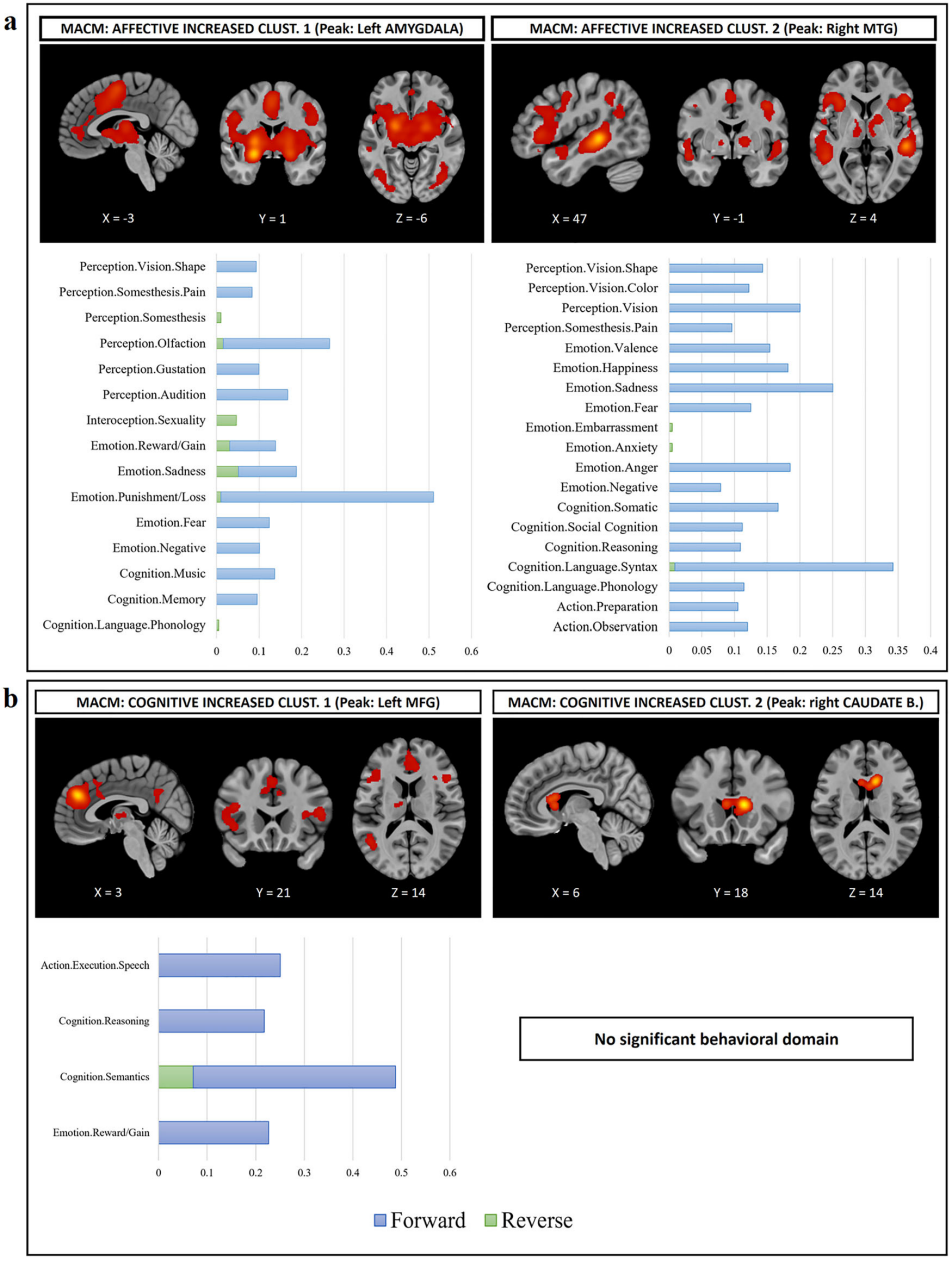
(a) Bar chart depicting meta‐analytical connectivity modeling (MACM) and functional decoding analysis, with two examples of affective‐increased clusters. (b) Two examples of cognitive‐increased clusters. Blue bars represent the probability of the forward inference analysis, while green bars depict a reversed inference probability.

MACM was applied using all clusters across the three experimental paradigms we found in the main ALE analysis. However, since the single cluster in cognitive increased included the anterior part of the lateral ventricle, which is a non‐neuronal segment, we decided to exclude this area, resulting in two sub‐clusters for the MACM analysis with the left MFG and right caudate body as respective peaks in each subcluster. After obtaining MACM outputs from all clusters in each experimental condition, we then performed the ALE analysis on those MACM outputs. For Subcluster 1 of cognitive increased, we found six MACM clusters, while there was only a single MACM cluster from Subcluster 2 of cognitive increased. For affective increased, there were five MACM clusters from Cluster 1, and seven MACM clusters from Cluster 2. Last, for affective decreased, there were three MACM clusters from Cluster 1, three MACM clusters from Cluster 2, and two MACM clusters from Cluster 3 (Table S4). In addition, we performed functional decoding based on the list of studies we found from the MACM analysis. The majority of psychological processes identified through functional decoding were associated with their corresponding task paradigm, although a smaller number of psychological functions were observed outside the designated task paradigm (Figures 3 and S2). For example, in affective increased Cluster 1 (Figure 3), we identified affective and interoceptive domains such as sadness, fear, and gustation, while also observing the presence of memory in this cluster.

### 3.3 Seed‐Based Resting‐State Functional Connectivity and Hierarchical Clustering

**FIGURE 4:**
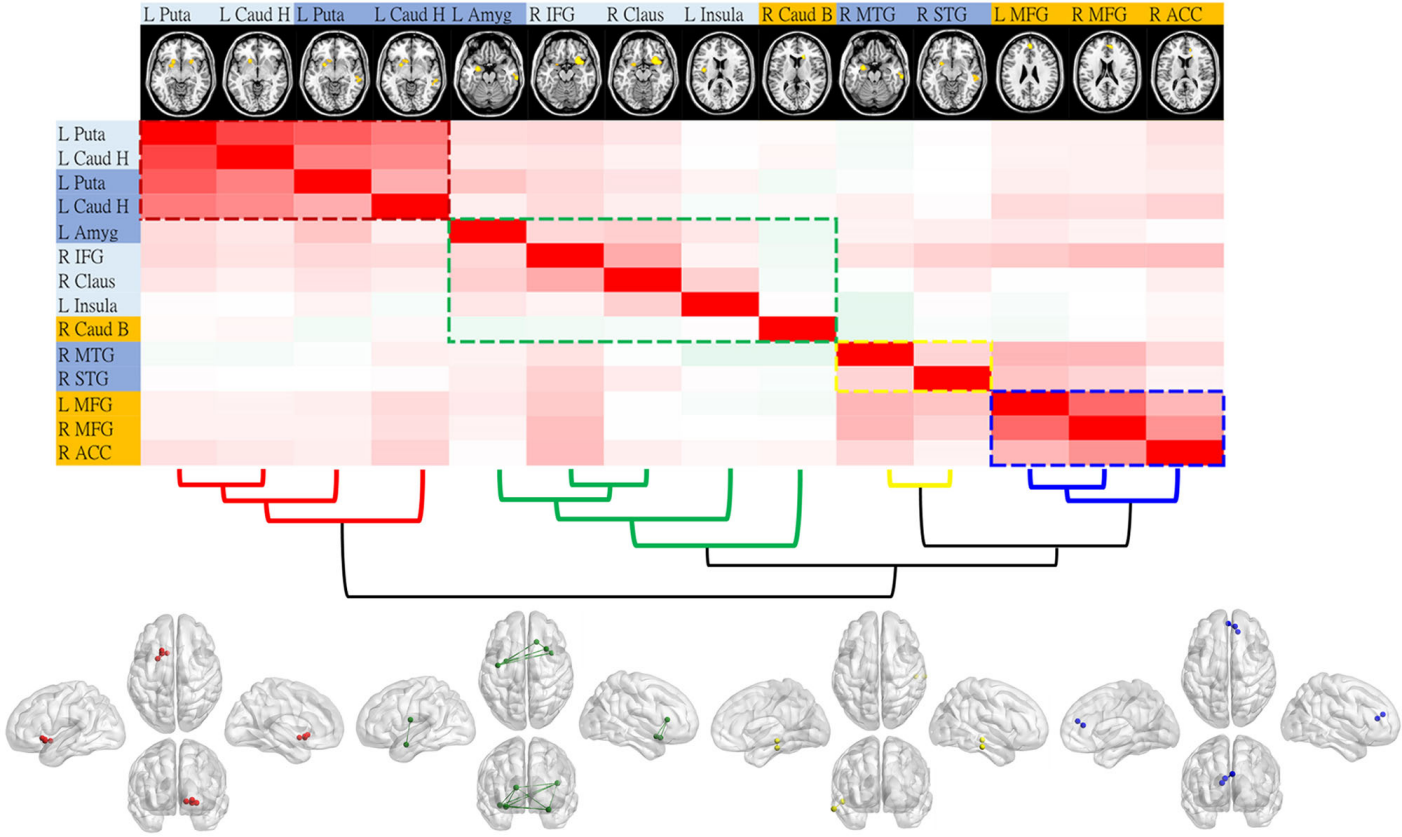
Clustering of the resting‐state functional connectivity analysis. Four clusters of connectivity illustrate similarities between each region of interest (ROI) within each cluster. Cluster 1 (red) depicts shared regions across affective domains (domain‐specific), whereas Cluster 2 includes ROIs across all paradigms (domain‐general). Clusters 2 (yellow) and 3 (blue) specifically represent affective‐increased and cognitive‐increased regions, respectively (domain‐specific). Note that the name of each ROI is within a colored cell, where dark blue represents affective‐increased, light blue represents affective‐decreased, and yellow represents cognitive‐increased ROIs.

RSFC was executed based on the average BOLD signal of 13 seeds (Figure 4) depicting whole‐brain connectivity given regions found in the main ALE analysis as seeds. We then clustered these 13 seed regions based on similarities of their RSFC patterns across participants using a Ward hierarchical analysis. Here, we identified four clusters of functional brain networks, where one cluster was domain‐general and the other three were domain‐specific (Figure 3). The first cluster included regions of interest (ROIs) specifically related to affective processing in general (i.e., affective increased‐ and affective decreased‐driven seeds), whereas the second cluster included ROIs from all experimental paradigms (i.e., domain‐general). On the other hand, the third and fourth clusters were domain‐specific, and respectively included affective‐increased and cognitive‐decreased ROIs.

### 3.4 Validation Analyses

For the jackknife sensitivity analysis, the majority of clusters in all experimental paradigms, except affective‐decreased Cluster 3, indicated consistent results with the output of the main ALE analysis after leaving one experiment out (Table S3). Likewise, the analysis of combining experiments within a paper also exhibited essentially similar findings as our main ALE analysis (Figures S2–S4).

## 4 Discussion

This study is the first meta‐analysis to investigate neural alterations in individuals with Type 2 diabetes using a task‐based paradigm. In contrast to prior meta‐analyses, our findings indicated various neural alterations in Type 2 diabetes depending on the category of the behavioral task, whereas previous meta‐analyses solely focused on resting‐state networks (e.g., DMN; Xia et al. 2017; Li et al. 2022; Yao et al. 2021). Our findings corroborate those that found aggregated GMV reductions globally across brain regions and locally in various brain regions, including regions outside resting‐state networks (Yao et al. 2021).

For the cognitive paradigm, we identified a cluster of task‐induced convergences of activation, with the MFG, part of medial frontal cortex (MFC), as the peak region. A comprehensive meta‐analysis that specifically examined the functional organization of the MFC (de la Vega et al. 2016) revealed its association with a wide range of psychological functions, particularly its middle and anterior regions. The middle part of the MFC was preferentially linked to cognitive control, pain perception, and affective processing, while the anterior part was associated with episodic memory, social cognition, and reward processing. It is noteworthy that the MFC is commonly reported to be co‐activated with the anterior cingulate cortex (ACC), as we also found in the current meta‐analysis. Co‐activation of both the MFC and ACC has been linked to various functions, including autonomic regulation (Critchley et al. 2003), autobiographical memory (Spreng and Grady 2010), mentalizing (Baumgartner et al. 2012), and valuation (Hare et al. 2009). Regarding Type 2 diabetes functional brain alterations, a recent study (Y. H. Chen et al. 2023) discovered increased co‐activation between the MFC and ACC during the execution of emotional Stroop tasks, despite a declining behavioral performance. This suggests poorer cognitive performance in Type 2 diabetes patients, although they have to expend more energy to complete such tasks, indicating MFC‐ACC co‐activation as a biomarker for emotion‐laden cognitive tasks. This was supported by studies demonstrating MFG‐ACC interactions during various cognitive tasks combined with emotional stimuli or involving individuals with emotion regulation difficulties, which hinder optimal cognitive performances (Etkin et al. 2011; Song et al. 2017).

In the affective paradigm, we observed both Type 2 diabetes‐related increases and decreases in brain activities. We acknowledge that the included affective studies utilized only food‐related tasks, which although is highly relevant to the challenges posed by Type 2 diabetes, such a uniform type of experiment might not fully represent the broader spectrum of “affective processes” themselves. However, despite the limited scope of the included affective experimental paradigm, our additional task‐based and task‐free connectivity analyses (MACM and RSFC) revealed neural patterns resembling known affective mechanisms. In this category, we found two clusters of increased activation, with peaks in the amygdala and MTG, and three clusters of decreased activation, with peaks in the IFG, insula, and putamen. Among these peaks, the amygdala, insula, and putamen are subcortical regions that are known as key regions in affective and motivational processes (Adolfi et al. 2017; Tang et al. 2012). Conversely, the MTG and IFG are typically associated with various psychological processes, including both affective and cognitive (Hartwigsen et al. 2019; Papeo et al. 2019).

It appears that the groups of clusters induced by these two independent paradigms were partly exclusive and partly overlapping. In line with this, results from the MACM and functional decoding analyses, based on the peak region of each cluster, revealed consistent patterns of the majority of behavioral domains associated with the cognitive clusters being related to cognitive functions, while clusters from the affective paradigm were linked to both cognitive and affective behavioral domains. Specifically, this aligns with the main ALE clusters, suggesting that clusters in which the amygdala, insula, and putamen were peak regions were more likely to be associated with affective‐related behavioral domains, whereas clusters in which the MTG and IFG were peak regions were more likely to be associated with both affective‐ and cognitive‐related behavioral domains. This pattern is plausible as affective tasks also often involve cognitive processes. Therefore, we may observe cognitive‐related regions in clusters induced by affective tasks, while affective‐related regions could also emerge to a lesser extent in clusters induced by cognitive tasks.

Furthermore, we conducted a task‐free analysis by exploring the resting‐state functional connectivity of the normal population using each peak region of every identified cluster as the seed region. We then clustered the connectivity patterns across all of these regions. Note that, as implemented in previous meta‐analysis (i.e., Gu et al. 2019), this additional step provides a complementary perspective via the intrinsic functional organization of the brain, that is independent of task‐related demands. Examining these connectivity patterns allows us to determine whether the regions identified in task‐based studies also co‐activate at rest, thus offering insights into their broader network‐level roles and potential dysfunctions in Type 2 diabetes. Our results revealed two affective‐related clusters, one cognitive‐related cluster, and one shared cluster. Corroborating our previous findings, the first affective‐related cluster (highlighted in red in Figure 3) comprised the putamen and caudate head, while the other affective cluster (highlighted in yellow in Figure 3) included the MTG and STG. In contrast, the cognitive cluster (highlighted in blue in Figure 3) consisted of the MFG and ACC, while the shared cluster (highlighted in green in Figure 3) encompassed the amygdala, IFG, claustrum, insula, and caudate body. These findings, again, underscore that neural alterations in Type 2 diabetes involve different brain networks that can be both domain‐specific and domain‐general in task‐based neuroimaging. Alterations in cognitive‐only brain networks may manifest as various cognitive deficits (Li et al. 2022). Conversely, alterations in affective‐only brain networks suggest that Type 2 diabetes patients may experience adverse emotional and motivational deficits, including problems related to appetite and reward mechanisms (e.g., ten Kulve et al. 2015). Interestingly, domain‐general networks may indicate challenges Type 2 diabetes patients face in emotion‐laden cognition, such as emotion regulation (e.g., Coccaro et al. 2022). These networks might also shed light on the emotional and motivational aspects and their interactions with executive function (Pessoa 2009) that are affected by hyperglycemia in Type 2 diabetes.

In general, our multilayer analysis showed consistent patterns of activation that were either domain‐specific or domain‐general. This outcome contributes to a broader understanding of brain alterations in Type 2 diabetes, as previous meta‐analyses primarily focused on alterations in resting‐state network areas, which are thought to underlie cognitive dysfunctions among Type 2 diabetes patients (e.g., Xia et al. 2017; Wu et al. 2023). In contrast, results of our current meta‐analysis emphasized distinct and shared functional domains that were consistent with findings from a previous multimodal meta‐analysis (Yao et al. 2021), which suggested that GMV reduction in Type 2 diabetes affects brain regions more globally, whereas functional alterations at rest tend to be localized within DMN regions. It is important to note that we do not contradict findings from the resting‐state‐based meta‐analysis. Instead, we found alteration of the MFG, a key DMN region, in the current meta‐analysis. However, in addition to this specific region, we provide additional insights from alterations in other loci, including regions associated with psychological functions that are not necessarily included in the DMN or any other resting‐state functional networks.

Despite providing novel insights into cognitive‐ and affective‐related brain alterations in Type 2 diabetes, some limitations of this study should be noted. First, the included studies provided unequal variations of tasks and contrasts between cognitive and affective paradigms, with more variations of tasks and contrasts in the cognitive paradigm. Here, the uniformity of contrasts would provide a more‐consistent convergence of activations while more‐variable contrasts would risk an eventual outcome of the convergence of activations. For instance, tasks like the *N*‐back and Stroop tasks, although both tap into similar cognitive processes (i.e., attention), may involve distinct neural pathways when executing the tasks. Hence, this unequal variation of contrasts could have been the underlying reason behind the unequal number of activation convergences between the cognitive and affective paradigms. However, the significant cluster of activation found in the cognitive paradigm might be indicative that such regions are hubs that typically alter activations in Type 2 diabetes and are implicated in deficits across various cognitive domains. Apart from that, only food‐related affective tasks were included in this analysis, potentially limiting the generalizability of findings to the broader domain of affective processes. However, it is important to note that regions identified in this paradigm (e.g., the amygdala and insula) are not exclusively associated with food‐related motivation or arousal, as they also play roles in generic emotional responses. These results are consistent with previous findings that linked emotional‐related brain alterations in Type 2 diabetes patients to various affective‐related issues at the clinical level, such as mood disorders (Ducat et al. 2014).

## 5 Conclusion

In summary, functional brain alterations in Type 2 diabetes occur in both common and distinct neural pathways depending on the task. While previous functional neuroimaging meta‐analyses emphasized neural alterations linked to decreased cognitive performance, the current meta‐analysis highlighted broader alterations that may reflect deficits in various psychological domains, including cognitive, emotional, and motivational aspects. These findings underscore longstanding issues related to mental health and quality of life among Type 2 diabetes patients (Coffey et al. 2002; Ducat et al. 2014), where, beyond cognitive deficits, patients may experience emotional and motivational challenges due to difficulties associated with long‐term Type 2 diabetes care. Therefore, it is crucial for families, caregivers, and society at large to understand the challenges faced by these patients. Psychological support at both the individual and community levels is necessary to address the various psychological problems that Type 2 diabetes may introduce into their lives.

## Author Contributions

**Valentino Marcel Tahamata**: conceptualization, data curation, formal analysis, methodology, writing – original draft. **Yang‐Teng Fan**: conceptualization, formal analysis, methodology. **Li Wei**: methodology, validation, project administration, writing – review and editing. **Yen‐Nung Lin**: validation, project administration, resources, writing – review and editing. **Roger Marcelo Martinez**: writing – review and editing, formal analysis, data curation. **Kah Kheng Goh**: writing – review and editing, validation, project administration. **Yu‐Chun Chen**: validation, project administration, writing – review and editing. **Chenyi Chen**: conceptualization, formal analysis, validation, writing – original draft, writing – review and editing.

## Ethics Statement

The authors have nothing to report.

## Consent

The authors have nothing to report.

## Conflicts of Interest

The authors declare no conflicts of interest.

### Peer Review

The peer review history for this article is available at https://publons.com/publon/10.1002/brb3.70438.

## Supporting information



Supporting Information

## Data Availability

The data that support the findings of this study are available in Nathan Kline Institute‐Rockland at http://fcon_1000.projects.nitrc.org/indi/enhanced/. These data were derived from the following resources available in the public domain: ‐ resting‐state data, https://fcon_1000.projects.nitrc.org/indi/enhanced/neurodata.html
