# Supplementary material for: Domain‐Specific and Domain‐General Functional Brain Alterations in Type 2 Diabetes Mellitus: A Task‐Based and Functional Connectivity Meta‐Analysis
Source: Brain Behav. 2025 Mar 31;15(4):e70438. doi: 10.1002/brb3.70438 (PMC11959097; doi:10.1002/brb3.70438)
Supplement: Supplementary file 1 — Supporting Information [file BRB3-15-e70438-s001.docx]

**SUPPLEMENTARY MATERIALS**

*Supplementary methods for the preprocessing of resting-state functional connectivity analysis*

RSFC analysis was performed to investigate the functional connectivity between all regions identified in the primary activation likelihood estimation (ALE) analysis. This study included resting-state fMRI data from 278 healthy adults, without current psychiatric or neurological diagnosis, sourced from the Enhanced Nathan Kline Institute – Rockland Sample. Resting-state fMRI images were acquired using a Siemens TimTrio 3T scanner with BOLD contrast and gradient-echo EPI pulse sequence. The imaging parameters were set as follows: TR, 2.5 s; TE, 30 ms; flip angle, 80; voxel size, 3.0 mm × 3.0 mm × 3.3 mm; slice number, 64 slices. The preprocessing of both fMRI and structural MRI data was carried out using the CONN-fMRI FC toolbox (v.22.a) (Whitfield-Gabrieli & Nieto-Castanon, 2012) and SPM8 from the Wellcome Trust Centre for Neuroimaging. The preprocessing steps included realignment and unwarping of functional images, slice-timing correction, coregistration with structural data, spatial normalization into the standard MNI space, outlier detection (ART-based scrubbing), and smoothing with a Gaussian kernel (FWHM of 8 mm). The structural data underwent segmentation into gray matter, white matter (WM), and cerebrospinal fluid (CSF), and were normalized using the default preprocessing pipeline. Nuisance covariates, such as signals from WM and CSF, as well as translational and rotational movement parameters, were regressed out using covariate regression analysis by CONN. The CompCor strategy was employed to reduce the impact of nuisance covariates, enhancing the accuracy of the analysis (Behzadi et al., 2007). Finally, band-pass filtering was applied with a frequency window of 0.008–0.09 Hz, a step known to enhance retest reliability.

**Table S1.** Average contribution of each contrasts to the significant clusters of activation

| **Cluster** | **Study** | **Task** | **Contrast** | **N Subj. (N Foci)** | **Average Contrib. (%)** |
| --- | --- | --- | --- | --- | --- |
| Cognitive Increased - 1 | Huang et al., (2015) | N-back | Higher > lower cognitive load | 18 (3) | 50 |
|  | Sun et al., (2017) | Iowa Gambling Task | Decision making > control condition | 18 (1) | 16.67 |
|  | Chen et al., (2023) | Stroop task | Incongruent > congruent condition | 48 (1) | 16.67 |
|  | Chen et al., (2023) | Stroop task | Incongruent > congruent condition | 78 (1) | 16.67 |
| Affective Increased - 1 | Farr & Mantzoros, (2019) | Food-related exposure | Higher > lower desirable food | 11 (1) | 9.09 |
|  | ten Kulve et al., (2016a) | Food-related exposure | Food > non-food | 20 (4) | 36.36 |
|  | van Bloemendaal et al., (2015) | Food-related exposure | Chocolate taste > tasteless | 16 (3) | 27.27 |
|  | Salem et al., (2021) | Food-related exposure | Food > non-food | 35 (3) | 27.27 |
| Affective Increased - 2 | Allen et al., (2016) | Food-related exposure | Food > non-food | 15 (2) | 22.22 |
|  | Allen et al., (2016) | Food-related exposure | Food > non-food | 57 (7) | 77.78 |
| Affective Decreased - 1 | ten Kulve et al., (2016b) | Food-related exposure | T2DM < HC | 40 (2) | 33.33 |
|  | van Bloemendaal et al., (2014) | Food-related exposure | T2DM < obese HC | 32 (2) | 33.33 |
|  | van Bloemendaal et al., (2015) | Food-related exposure | Placebo < EXE | 16 (2) | 33.33 |
| Affective Decreased - 2 | Frank et al., (2016) | Food-related exposure | Nonsurgical < RGYB | 24 (1) | 33.33 |
|  | van Bloemendaal et al., (2015) | Food-related exposure | Placebo < EXE | 16 (2) | 66.67 |
| Affective Decreased - 3 | van Bloemendaal et al., (2015) | Food-related exposure | Placebo < EXE | 16 (4) | 100 |

**Notes:** T2DM = type 2 diabetes mellitus; HC = healthy control; EXE = exenatide; RGYB = Roux-en-Y Gastric Bypass

**Table S2.** Overall MACM outcomes after ALE

| **Cluster** | **N Contrasts (N Foci)** | **N Subj.** | | **N Experiment (ALE)** | **Cluster Peak** | | | **N Foci (ALE)** | **Foci Average Contribution (%)** | |  |
| --- | --- | --- | --- | --- | --- | --- | --- | --- | --- | --- | --- |
| Cognitive Increased - 1 | 71 (841) | 1235 | | 70 (Cluster 1) | L MFG | | | 141 | 16.77 | |  |
|  |  |  | | 22 (Cluster 2) | L Insula | | | 32 | 3.80 | |  |
|  |  |  | | 17 (Cluster 3) | R Thalamus | | | 22 | 2.66 | |  |
|  |  |  | | 15 (Cluster4) | R IFG | | | 16 | 1.90 | |  |
|  |  |  | | 14 (Cluster 5) | L CG | | | 15 | 1.78 | |  |
|  |  |  | | 11 (Cluster6) | L MTG | | | 13 | 1.55 | |  |
| Cognitive Increased - 2 | 4 (25) | 63 | | 4 (Cluster1) | Caudate Body | | | 7 | 28 | |  |
| Affective Increased - 1 | 308 (4609) | 5542 | | 308 (Cluster 1) | L Amygdala | | | 1938 | 42.05 | |  |
|  |  |  | | 148 (Cluster 2) | L MFG | | | 294 | 6.38 | |  |
|  |  |  | | 96 (Cluster 3) | L Fusif. G. | | | 160 | 3.47 | |  |
|  |  |  | | 94 (Cluster 4) | R Fusif. G. | | | 141 | 3.06 | |  |
|  |  |  | | 53 (Cluster 5) | R STG | | | 61 | 1.32 | |  |
| Affective Increased - 2 | 104 (1707) | 1959 | | 104 (Cluster 1) | R STG | | | 223 | 13.06 | |  |
|  |  |  | | 75 (Cluster 2) | L MTG | | | 176 | 10.31 | |  |
|  |  |  | | 63 (Cluster 3) | R IFG | | | 127 | 7.44 | |  |
|  |  |  | | 61 (Cluster 4) | L Insula | | | 113 | 6.62 | |  |
|  |  |  | | 33 (Cluster 5) | L MFG | | | 59 | 3.46 | |  |
|  |  |  | | 20 (Cluster 6) | R Thalamus | | | 34 | 1.99 | |  |
|  |  |  | | 25 (Cluster 7) | L VPLS | | | 27 | 1.58 | |  |
| Affective Decreased - 1 | 192 (2961) | 3497 | | 192 (Cluster 1) | R Putamen | | | 1001 | 33.81 | |  |
|  |  |  | | 121 (Cluster 2) | L CG | | | 282 | 9.53 | |  |
|  |  |  | | 31 (Cluster 3) | R Insula | | | 40 | 1.35 | |  |
| Affective Decreased - 2 | 104 (1367) | 1496 | | 104 (Cluster 1) | L Insula | | | 601 | 43.96 | |  |
|  |  |  | | 36 (Cluster 2) | L CG | | | 55 | 4.02 | |  |
|  |  |  | | 18 (Cluster 3) | R Culmen | | | 20 | 1.46 | |  |
| Affective Decreased - 3 | 89 (1310) | 1652 | | 89 (Cluster 1) | L Putamen | | | 476 | 36.34 | |  |
|  |  |  |  | 21 (Cluster 2) | | L CG | 31 | | | 2.37 | |

*Note*: MFG = medial frontal gyrus, IFG = inferior frontal gyrus, CG = cingulate gyrus, MTG = middle temporal gyrus, STG = superior temporal gyrus, VPLS = ventral posterior lateral nucleus.

**Table S3.** Jackknife analysis.

| **Cluster** | **Laterality** | **Brain Regions** | **BA** | **MNI** | | | **Jackknife** |  |  |
| --- | --- | --- | --- | --- | --- | --- | --- | --- | --- |
|  |  |  |  | **x** | **y** | **z** |  |  |  |
| ***Cognitive Increased Activity*** | | |  |  |  |  |  |  |  |
| 1 | L | Medial Frontal Gyrus | 9 | 0 | 50 | 26 | 7/10 |  |  |
|  | R | Medial Frontal Gyrus | 9 | 10 | 46 | 20 | 7/10 |  |  |
|  | R | Caudate Body | / | 16 | 26 | 10 | 7/10 |  |  |
|  | R | Anterior Cingulate Cortex | 32 | 14 | 38 | 14 | 7/10 |  |  |
| ***Cognitive Decreased Activity*** | | |  |  |  |  |  |  |  |
| None |  |  |  |  |  |  |  |  |  |
| ***Affective Increased Activity*** | | |  |  |  |  |  |  |  |
| 1 | L | Amygdala | / | -28 | -2 | -20 | 8/11 |  |  |
|  | L | Putamen | / | -24 | 6 | -8 | 8/11 |  |  |
|  | L | Caudate Head | / | -10 | 14 | -6 | 8/11 |  |  |
|  | L | Putamen | / | -20 | 12 | 0 | 8/11 |  |  |
|  | L | Putamen | / | -18 | 16 | -4 | 8/11 |  |  |
| 2 | R | Middle Temporal Gyrus | 21 | 66 | -24 | -20 | 9/11 |  |  |
|  | R | Middle Temporal Gyrus | 21 | 60 | -32 | -6 | 9/11 |  |  |
|  | R | Superior Temporal Gyrus | 22 | 54 | -24 | -10 | 9/11 |  |  |
|  | R | Middle Temporal Gyrus | 21 | 56 | -34 | -8 | 9/11 |  |  |
|  | R | Middle Temporal Gyrus | 21 | 60 | -16 | -20 | 9/11 |  |  |
|  | R | Middle Temporal Gyrus | / | 54 | -34 | -2 | 9/11 |  |  |
|  | R | Middle Temporal Gyrus | 22 | 52 | -40 | -4 | 9/11 |  |  |
| ***Affective Decreased Activity*** | | |  |  |  |  |  |  |  |
| 1 | R | Inferior Frontal Gyrus | 47 | 30 | 16 | -16 | 3/5 |  |  |
|  | R | Claustrum | / | 38 | 10 | -14 | 3/5 |  |  |
| 2 | L | Insula | 13 | -40 | -8 | 16 | 3/5 |  |  |
|  | L | Insula | 13 | -44 | -12 | 8 | 3/5 |  |  |
|  | L | Insula | 13 | -44 | -18 | 8 | 3/5 |  |  |
| 3 | L | Putamen | / | -18 | 14 | -8 | 1/5 |  |  |
|  | L | Caudate Head | / | -18 | 18 | -4 | 1/5 |  |  |
|  | L | Putamen | / | -24 | 5 | -11 | 1/5 |  |  |
|  | L | Putamen | / | -20 | 5 | -11 | 1/5 |  |  |


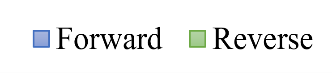

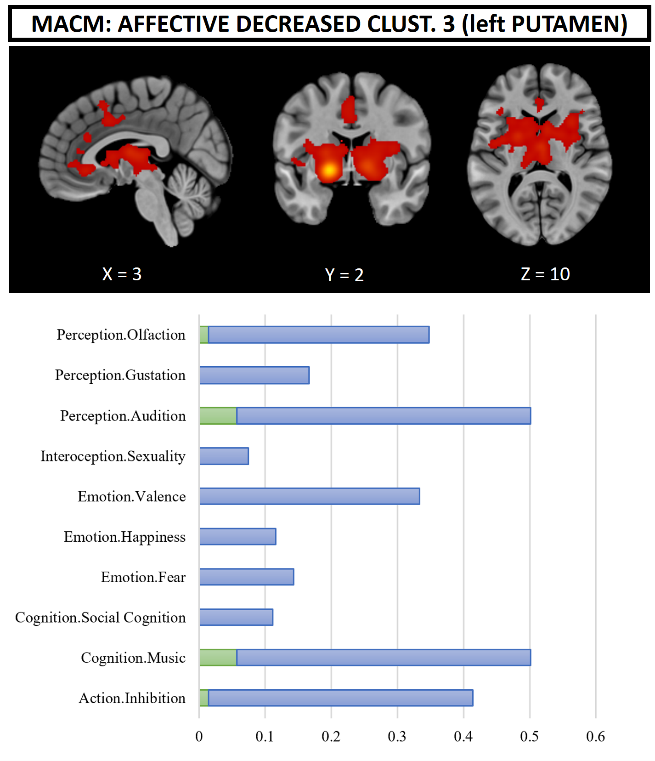

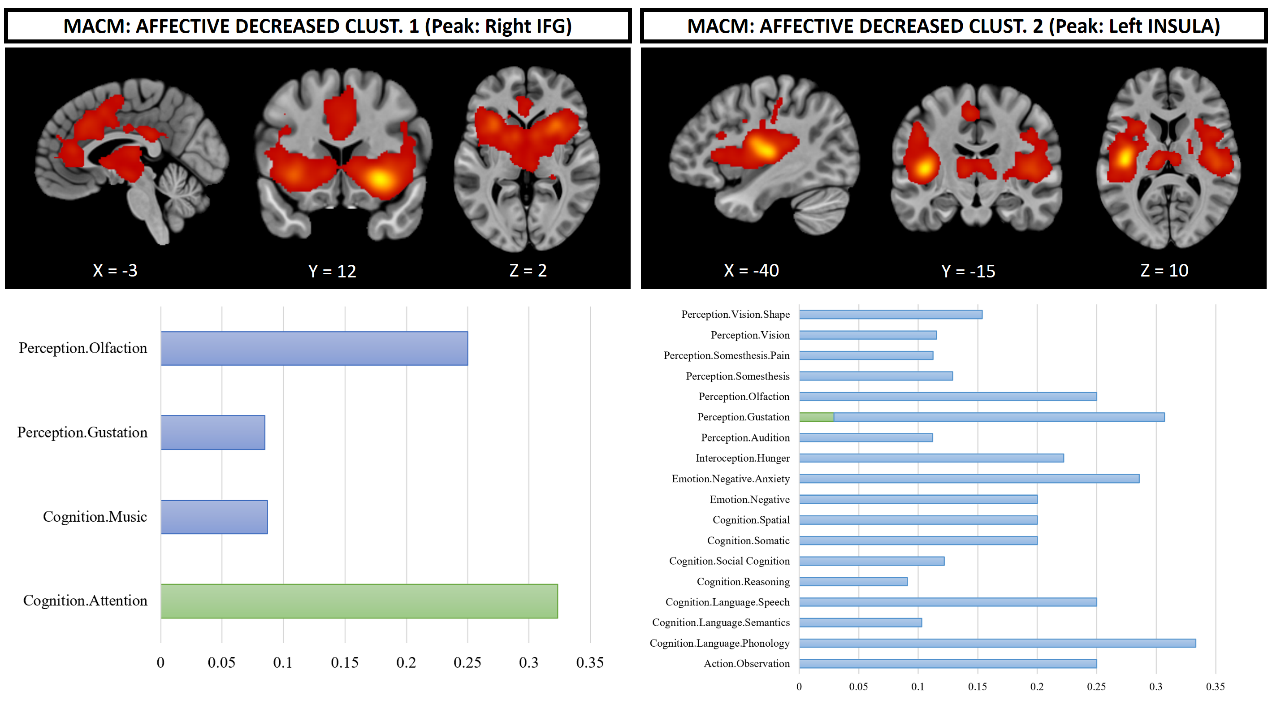


**Figure S1.** Bar chart depicting MACM and functional decoding analysis, three examples from affective-decreased clusters. Blue bars represent the probability of forward inference analysis, while green bars depict reversed inference probability.


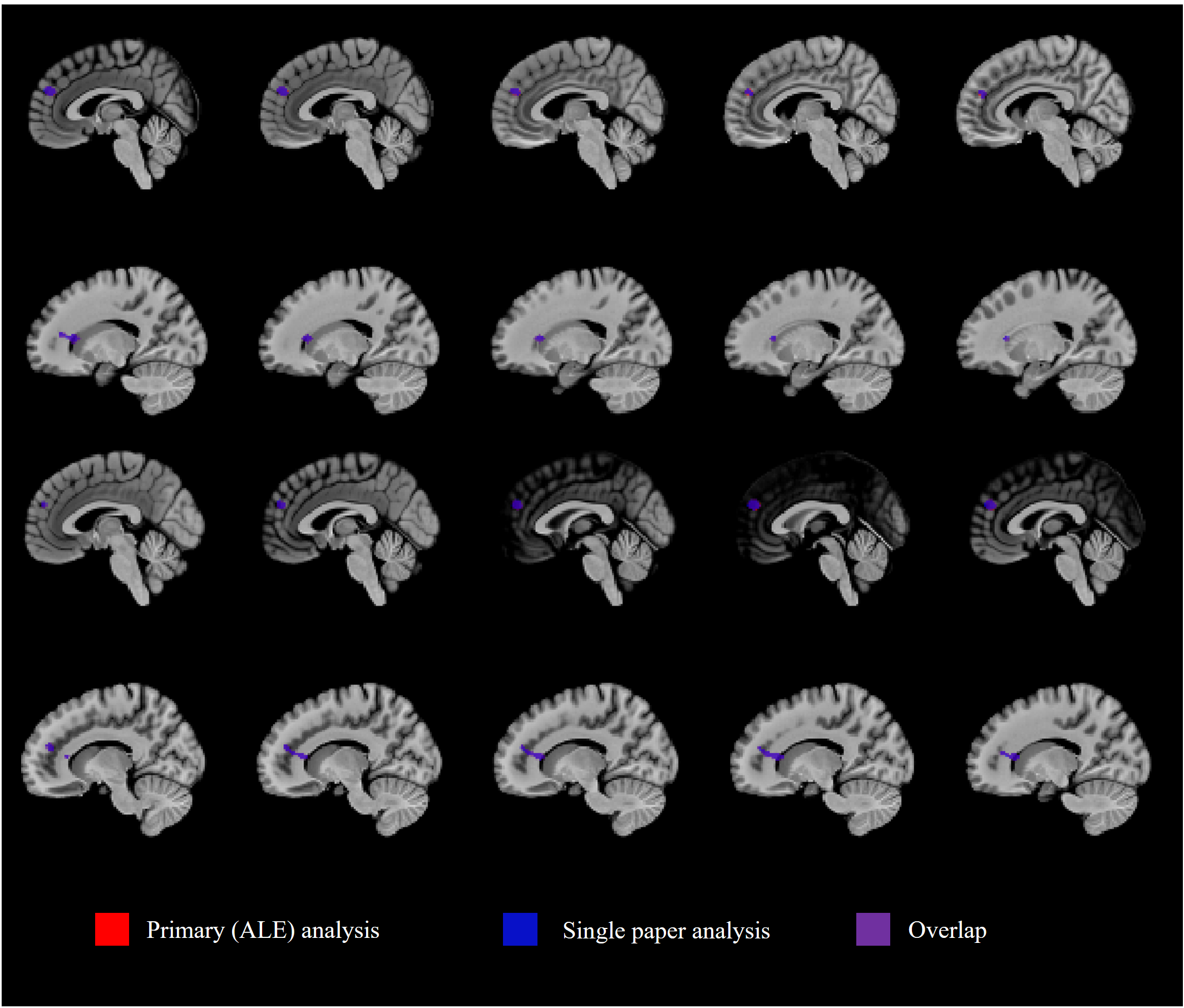


**Figure S2.** Single-paper analysis on cognitive increased


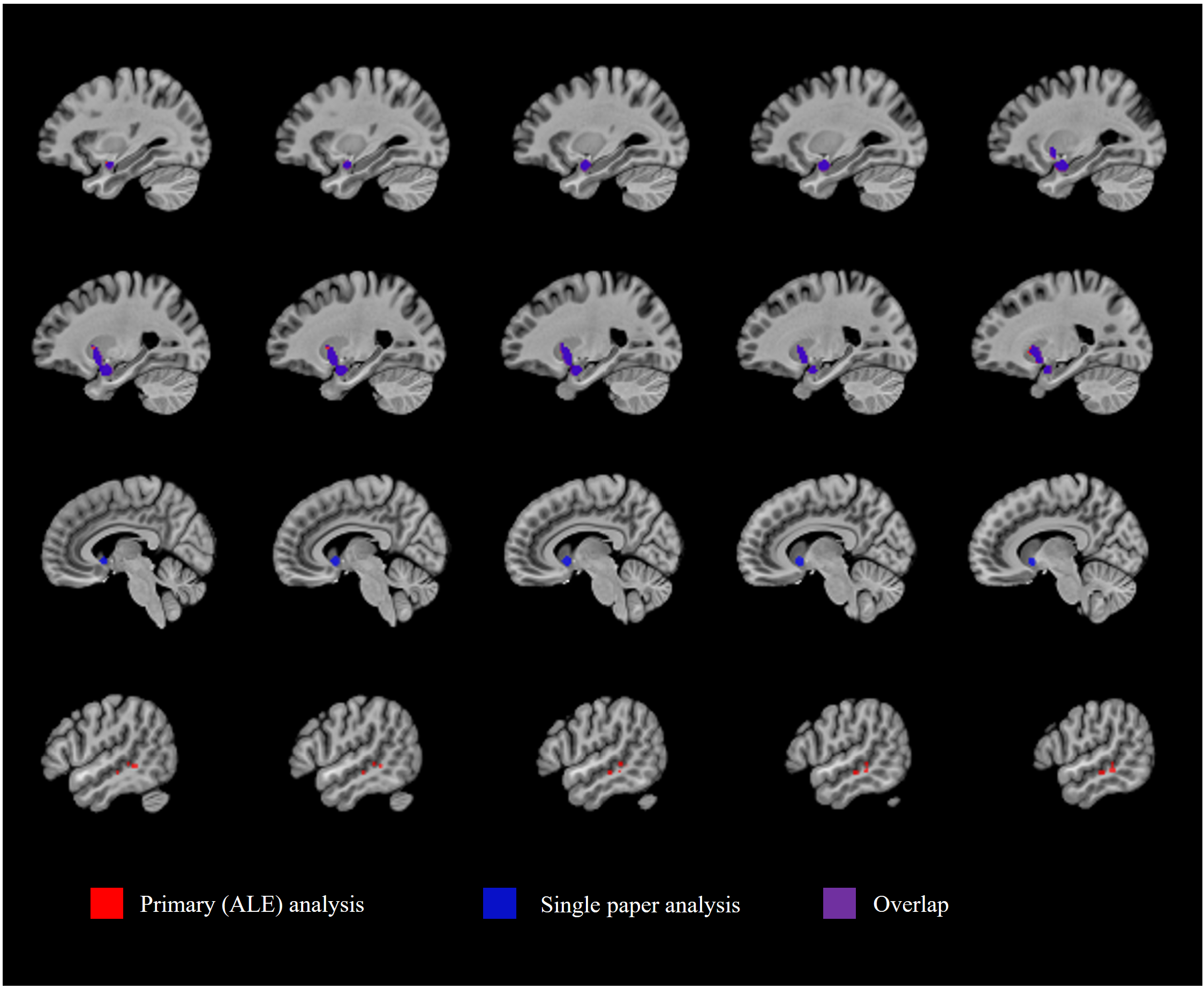


**Figure S3.** Single-paper analysis on affective increased


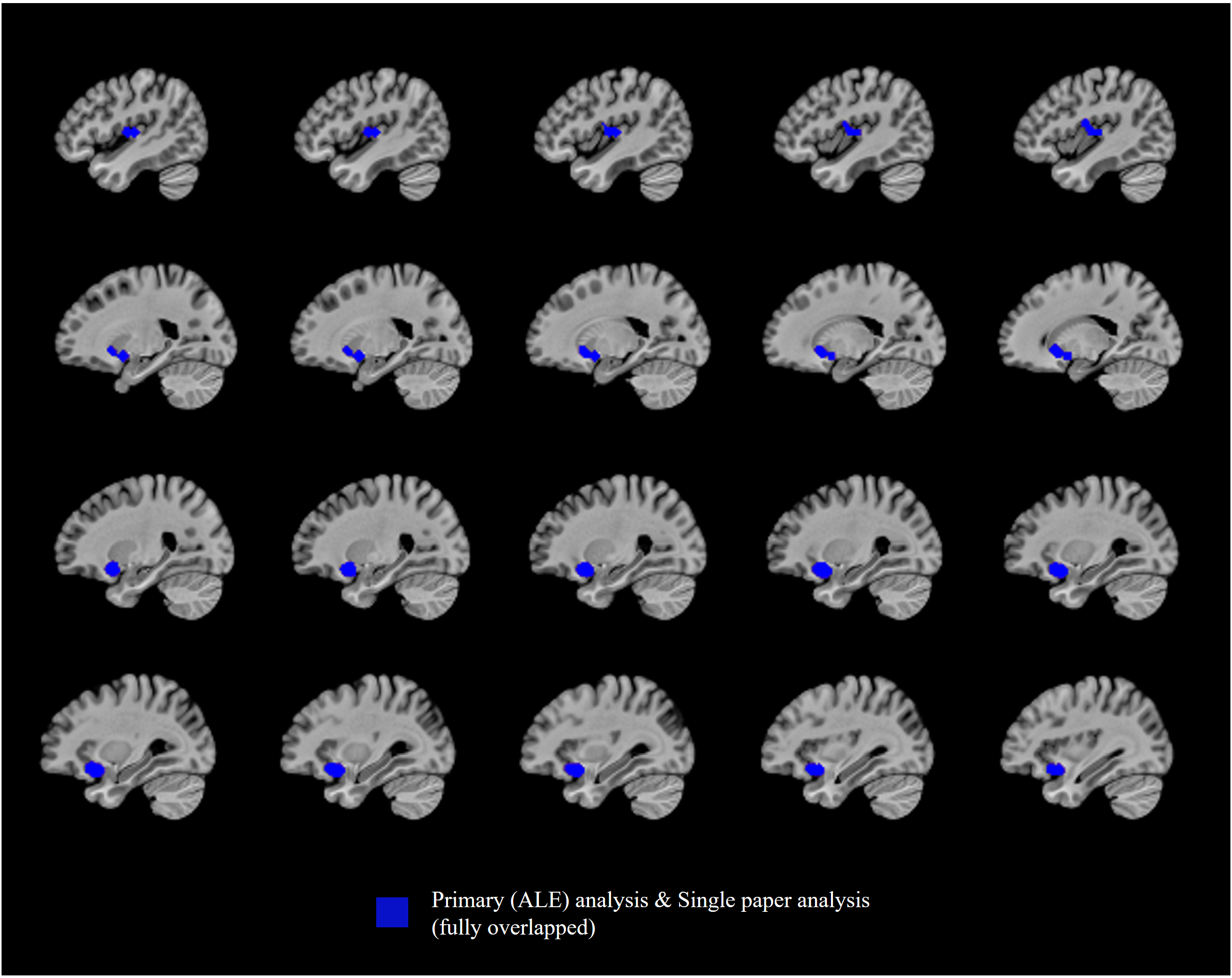


**Figure S4.** Single-paper analysis on affective decreased

**Reference**

1. Allen HA, Chambers A, Blissett J, Chechlacz M, Barrett T, Higgs S, Nouwen A. Relationship between parental feeding practices and neural responses to food cues in adolescents. PLoS One. 2016;11(8):e0157037.
2. Backeström A, Papadopoulos K, Eriksson S, Olsson T, Andersson M, Blennow K, ... & Rolandsson O. Acute hyperglycaemia leads to altered frontal lobe brain activity and reduced working memory in type 2 diabetes. PLoS One. 2021;16(3):e0247753.
3. Chechlacz M, Rotshtein P, Klamer S, Porubska K, Higgs S, Booth D, ... & Nouwen A. Diabetes dietary management alters responses to food pictures in brain regions associated with motivation and emotion: a functional magnetic resonance imaging study. Diabetologia. 2009;52:524-533.
4. Duarte JV, Pereira JM, Quendera B, Raimundo M, Moreno C, Gomes L, ... & Castelo-Branco M. Early disrupted neurovascular coupling and changed event level hemodynamic response function in type 2 diabetes: an fMRI study. J Cereb Blood Flow Metab. 2015;35(10):1671-1680.
5. Farr, O. M., & Mantzoros, C. S. Obese individuals with type 2 diabetes demonstrate decreased activation of the salience‐related insula and increased activation of the emotion/salience‐related amygdala to visual food cues compared to non‐obese individuals with diabetes: a preliminary study. Diabetes Obes Metab. 2018;20(10):2500-2503.
6. Frank, S., Heinze, J. M., Fritsche, A., Linder, K., von Feilitzsch, M., Königsrainer, A., ... & Preissl, H. Neuronal food reward activity in patients with type 2 diabetes with improved glycemic control after bariatric surgery. Diabetes Care. 2016;39(8):1311-1317.
7. Huang, R. R., Jia, B. H., Xie, L., Ma, S. H., Yin, J. J., Sun, Z. B., ... & Luo, D. X. Spatial working memory impairment in primary onset middle‐age type 2 diabetes mellitus: An ethology and BOLD‐fMRI study. J. Magn Reson Imaging. 2016;43(1):75-87.
8. Marder, T. J., Flores, V. L., Bolo, N. R., Hoogenboom, W. S., Simonson, D. C., Jacobson, A. M., ... & Musen, G. Task-induced brain activity patterns in type 2 diabetes: a potential biomarker for cognitive decline. Diabetes. 2014;63(9):3112-3119.
9. McDermott, K. D., Williams, S. E., Espeland, M. A., Erickson, K., Neiberg, R., Wadden, T. A., ... & Action for Health in Diabetes Brain Magnetic Resonance Imaging (Look AHEAD Brain) Ancillary Study Research Group. Impact of intensive lifestyle intervention on neural food cue reactivity: action for health in diabetes brain ancillary study. Obesity. 2019;27(7):1076-1084.
10. Sun, D. M., Ma, Y., Sun, Z. B., Xie, L., Huang, J. Z., Chen, W. S., ... & Ma, S. H. Decision-making in primary onset middle-age type 2 diabetes mellitus: A BOLD-fMRI study. Sci Rep. 2017;7(1):10246.
11. ten Kulve, J. S., Veltman, D. J., van Bloemendaal, L., Barkhof, F., Drent, M. L., Diamant, M., & IJzerman, R. G. Liraglutide reduces CNS activation in response to visual food cues only after short-term treatment in patients with type 2 diabetes. Diabetes Care. 2016a;39(2):214-221.
12. ten Kulve, J. S., Veltman, D. J., van Bloemendaal, L., Groot, P. F., Ruhe, H. G., Barkhof, F., ... & Ijzerman, R. G. Endogenous GLP1 and GLP1 analogue alter CNS responses to palatable food consumption. J Endocrinol. 2016b;229(1):1-12.
13. van Bloemendaal, L., IJzerman, R. G., Ten Kulve, J. S., Barkhof, F., Konrad, R. J., Drent, M. L., ... & Diamant, M. GLP-1 receptor activation modulates appetite- and reward-related brain areas in humans. Diabetes. 2014;63(12):4186-4196.
14. van Bloemendaal, L., Veltman, D. J., ten Kulve, J. S., Groot, P. F., Ruhé, H. G., Barkhof, F., ... & IJzerman, R. G. Brain reward‐system activation in response to anticipation and consumption of palatable food is altered by glucagon‐like peptide‐1 receptor activation in humans. Diabetes Obes Metab. 2015;17(9):878-886.
15. Wood, A. G., Chen, J., Moran, C., Phan, T., Beare, R., Cooper, K., ... & Srikanth, V. Brain activation during memory encoding in type 2 diabetes mellitus: a discordant twin pair study. Journal of diabetes research. 2016; 3978428.
16. Zhang, Y., Lu, S., Liu, C., Zhang, H., Zhou, X., Ni, C., ... & Zhang, Q. Altered brain activation and functional connectivity in working memory related networks in patients with type 2 diabetes: An ICA-based analysis. Sci Rep. 2016;6(1):23767.
17. Salem, V., Demetriou, L., Behary, P., Alexiadou, K., Scholtz, S., Tharakan, G., ... & Tan, T. M. M. Weight loss by low-calorie diet versus gastric bypass surgery in people with diabetes results in divergent brain activation patterns: a functional MRI study. Diabetes Care. 2021;44(8):1842-1851.
18. Chen YH, Chen C, Jian HY, Chen YC, Fan YT, Yang CY, Cheng Y. The neural correlates of emotional conflict monitoring as an early manifestation of affective and cognitive declines in persons with Type 2 diabetes. Brain Commun. 2023;5(1):fcad022.
19. Baker, L. D., Cross, D. J., Minoshima, S., Belongia, D., Watson, G. S., & Craft, S. Insulin resistance and Alzheimer-like reductions in regional cerebral glucose metabolism for cognitively normal adults with prediabetes or early type 2 diabetes. Arch. Neurol. 2011;68(1):51-57.
20. Whitfield-Gabrieli, S., & Nieto-Castanon, A. Conn: A functional connectivity toolbox for correlated and anticorrelated brain networks. Brain Connect. 2012;2(3):125-141.
21. Behzadi Y, Restom K, Liau J, Liu TT. A component-based noise correction method (CompCor) for BOLD and perfusion-based fMRI. Neuroimage. 2007;37(1):90-101.

**Note:** references no. 1-19 (in red) are included studies.
